# VALIDATION OF THE ENVIRONMENTAL AUDIT SCORING EVALUATION (EASE) TOOL FOR LTC HOUSEHOLDS

**DOI:** 10.1093/geroni/igac059.3084

**Published:** 2022-12-20

**Authors:** Migette Kaup, Margaret Calkins, Adam Davey, Robert Wrublowsky, Ashlyn Zachgo, Samantha Gibson, Madison Parker

**Affiliations:** Kansas State University, Manhattan, Kansas, United States; IDEAS Institute, Cleveland Hts, Ohio, United States; University of Delaware, Newark, Delaware, United States; MMP Architects, Inc., Winnipeg, Manitoba, Canada; Kansas State University, Manhattan, Kansas, United States; University of Kansas, Lawrence, Kansas, United States; Wheat State Manor, Whitewater, Kansas, United States

## Abstract

This poster will share the results from a research initiative funded by the National Institutes of Health to assess the validity of the Environmental Audit Scoring Evaluation (EASE) tool in its ability to distinguish between different types of skilled care models based on the environmental and operational practices that can be observed and documented. The EASE tool was compared against three existing tools; PEAP, TESS-NH, and EAT-HC. Twenty-eight living areas in nursing homes across the state of Kansas identified as a traditional, household, or hybrid model were observed. The scores of the EASE were compared against the scores of three existing tools in order to evaluate its construct validity. The EAT-HC was most closely related to the EASE, with an R-value of 0.8817. The PEAP and the TESS-NH were less correlated to the EASE, with R-values of 0.8175 and 0.7097, respectively. Results found that the EASE was able to distinguish between traditional and homelike settings, though it could not identify hybrid models with a high degree of certainty. The analysis of variance between homelike and traditional homes was significant at 0.016, while the variance between homelike and hybrid and between hybrid and traditional were not significant. Inter-rater reliability of the EASE was consistently high (.96 and above). The outcomes demonstrated the EASE tool was able to assess the homelike characteristics of the environment of nursing homes better than or equally as well as previously validated tools.

